# A Hierarchical Multi-Resolution Self-Supervised Framework for High-Fidelity 3D Face Reconstruction Using Learnable Gabor-Aware Texture Modeling

**DOI:** 10.3390/jimaging12010026

**Published:** 2026-01-05

**Authors:** Pichet Mareo, Rerkchai Fooprateepsiri

**Affiliations:** 1 Business Administration and Information Technology Faculty, Rajamangala University of Technology Tawan-ok, Bangkok 10400, Thailand; 2 Innovative Education and Lifelong Learning Institute, Rajamangala University of Technology Tawan-ok, Chonburi 20110, Thailand

**Keywords:** 3D face reconstruction, multi-resolution modeling, self-supervised learning, gabor-aware texture enhancement, wavelet-based detail perception, Markov random field loss, high-frequency geometric details

## Abstract

High-fidelity 3D face reconstruction from a single image is challenging, owing to the inherently ambiguous depth cues and the strong entanglement of multi-scale facial textures. In this regard, we propose a hierarchical multi-resolution self-supervised framework (HMR-Framework), which reconstructs coarse-, medium-, and fine-scale facial geometry progressively through a unified pipeline. A coarse geometric prior is first estimated via 3D morphable model regression, followed by medium-scale refinement using a vertex deformation map constrained by a global–local Markov random field loss to preserve structural coherence. In order to improve fine-scale fidelity, a learnable Gabor-aware texture enhancement module has been proposed to decouple spatial–frequency information and thus improve sensitivity for high-frequency facial attributes. Additionally, we employ a wavelet-based detail perception loss to preserve the edge-aware texture features while mitigating noise commonly observed in in-the-wild images. Extensive qualitative and quantitative evaluation of benchmark datasets indicate that the proposed framework provides better fine-detail reconstruction than existing state-of-the-art methods, while maintaining robustness over pose variations. Notably, the hierarchical design increases semantic consistency across multiple geometric scales, providing a functional solution for high-fidelity 3D face reconstruction from monocular images.

## 1. Introduction

The use of high-fidelity 3D facial representations has gained prominence in widely publicized human–machine interaction applications such as facial recognition and analysis [1,2,3,4], intelligent medical systems [5,6], and facial animation [7,8]. The most well-known parametric 3D facial model, which is the 3D morphable model (3DMM), was proposed in [9], and its advanced geometric representation property allows the efficient image-based reconstruction of 3D facial geometry [10,11]. However, the recoverability of fine-grained 3D facial structures from a 2D image is still hindered by limited depth cues and spatial information, primarily inherent in 2D images. So far, progress has been made by using a deep learning methodology of regressing 3DMM parameters to improve reconstruction quality. The authors of [12] generated a weakly supervised method where image-level and perceptual losses were computed to recover facial geometry between the input and reconstructed images. Similarly, the authors of [13] proposed a self-supervised framework, which ensures shape consistency across viewpoint, illumination, and occlusion, allowing a more exact recognition of geometric attributes. While these methods can reconstruct coarse facial geometry, they fail to recover high-fidelity, fine-scale features like crow’s feet and subtle skin microstructures. To overcome this limitation, much research has focused on extracting detailed facial information via a prediction of detail maps, which has achieved significant performance gains [14,15,16].

**Figure 1:**
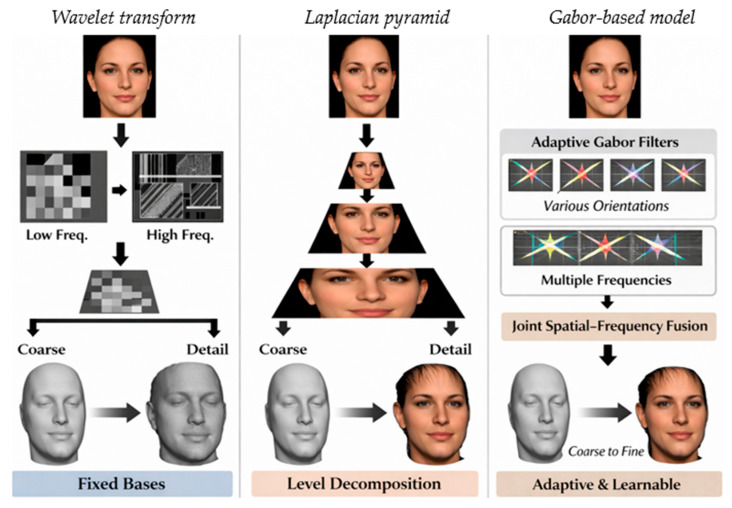
Conceptual illustration comparing Gabor-based spatial–frequency decoupling with alternative multi-scale representations, including wavelet transforms and Laplacian pyramids. Unlike fixed basis decompositions, the learnable Gabor-aware module provides adaptive orientation and frequency selectivity, enabling joint spatial–frequency modeling tailored to fine-scale facial detail reconstruction.

As illustrated in Figure 1, existing approaches exhibit distinct differences in their strategies for modeling and recovering facial geometry across multiple spatial scales, which directly influence their reconstruction capabilities and limitations. Each method has its limitations, however. The authors of [15] required high-quality scans during training, which limited generalization to in-the-wild imagery. The authors of [16] developed UV displacement maps to detect expression-related wrinkles and obtained much better recovery of fine-scale detail; however, they did not restore medium-scale geometric structures like dimples and deep nasolabial folds. The authors of [14], for example, made a prediction for displacement depth maps to reconstruct medium-scale features better, although fine-scale geometry was still suboptimal. Taken together, these shortcomings indicate the challenges of recovering high-dimensional multi-scale geometric features on a single detail map. In this study, a hierarchical multi-resolution framework based-on self-supervised learning (HMR-Framework) is proposed to address these problems. The framework is structured in a three-stage architecture focused on reconstructing large-, medium-, and fine-scale geometric facial information and trained at each stage using a self-supervised strategy. At the first stage, a 3DMM is used as the geometric prior, in which UV-space detail maps are developed slowly toward the fine detail of medium size- and fine size-scaled surfaces.

Since UV texture maps comprise simultaneously multiple multi-scale and multi-frequency levels of information, directly inputting these results into a neural network would hinder the learning of ultra-high frequency structures [17]. This implies that a robust decoupling mechanism is needed to decouple high-frequency textures and model them separately. Inspired by recent evidence of the high spatial-frequency texture extraction power of Gabor filters on fine-grained recognition tasks [18], a learnable Gabor-aware texture enhancement module is proposed. By coupling the learnable Gabor with a couple of constraints, this module promotes co-decoupling between medium- and fine-scale textures (spatial and frequency). These high-frequency features are fused with the UV texture map and computed by a CNN, facilitating the accurate reconstruction of fine-scale facial structures. Due to the serious impacts of skin imperfections and noise in these in-the-wild facial images, we developed detail perception loss (DPLoss) based on wavelet transform to mitigate its negative effects. Wavelet transform [19] allows facial textures to be encoded as sparse coefficients in the wavelet domain, providing noise robustness with the stability provided by preserving important structural information for performing fine-detail reconstructions. Additionally, fine-grained facial features are mainly centralized near the eye and mouth areas; because of that, they are constrained in isolation [20]. A Markov random field loss [21] is developed to enhance the spatial coherence of global and local representations of features. The main contribution of the work presented in this paper is as follows:

- We propose to model multi-level geometric facial features in the hierarchical mode using the hierarchical multi-resolution framework based on self-supervised learning (HMR-Framework).
- A learnable Gabor-aware texture enhancement module can be proposed to enhance fine-scale detail reconstruction by a joint spatial-frequency decoupling. This module constitutes the first incorporation of a learnable Gabor-based convolutional layer into the 3D face reconstruction pipeline to enable adaptive learning of high-frequency detail parameters.
- Global and local Markov random field loss (GL-MRFLoss) and detail perception loss (DPLoss) are proposed to deliver the global–local perceptual guidance and to ensure the structural properties of fine-scale facial features.

Despite recent advances in single-image 3D face reconstruction, existing approaches remain limited in their ability to jointly recover medium- and fine-scale geometric details under a unified self-supervised framework. Methods relying on a single displacement or detail map often suffer from scale entanglement, leading to either over-smoothed reconstructions or unstable high-frequency artifacts. To address these limitations, a hierarchical multi-resolution self-supervised framework (HMR-Framework) is proposed, in which facial geometry is progressively reconstructed from coarse to fine scales. By explicitly decoupling medium- and high-frequency information and introducing spatial–frequency-aware modeling, the proposed approach enables structurally consistent and perceptually faithful 3D facial reconstruction from a single monocular image.

## 2. Literature Review

### 2.1. Morphable Model-Based 3D Face Reconstruction (3DMM)

3DMM is considered a reliable geometric prior for 3D face reconstruction, allowing an estimation of 3D face geometry from a single 2D image in a short time. A variety of deep learning methods [22,23] applied deep neural network architectures to directly regress the 3DMM coefficients and obtained impressive precision. As pseudo-ground-truth labels, 3D facial models gained by optimization-guided procedures were implemented in [24,25] to be able to supervise the learning of the convolutional neural networks. The authors of [26] optimized reconstruction accuracy by using synthetic facial images with their 3D scans as training data. While these learning-based techniques made remarkable progress, their performance was limited by the unreliable nature of the training labels, which created data discrepancy between the reconstruction findings and the images involved. Because there are few paired 2D–3D facial datasets out there, self-supervised learning has emerged as an invaluable research avenue. In [27], a modulation-based deformation model was proposed for the self-supervised learning of shape correspondences between images and deformable meshes, leading to higher geometric fidelity. Ref. [12] suggested a weakly supervised hybrid loss to direct reconstruction by comparing the input and reconstructed images. The authors of [28] applied 2D facial landmark heatmaps and constructed four self-supervised learning methods to achieve relatively higher accuracy in 3DMM reconstruction. The study in [29] utilized the cyclic consistency of dynamic 3D facial features as a signal for in-the-wild reconstruction in a self-supervised manner. The authors of [30] decomposed facial features into identity and expression and modeled them separately; this way, the robustness of 3DMM can be improved with expression differences. However, even with the aforementioned progress in facial representation, the expressive ability of the original 3DMM is only restricted to the problem of producing coarse 3D face shapes without fine-grained information. To address this limitation, some extensions have been suggested. In [31], a nonlinear 3DMM was proposed and has a wider resolution to improve the visualization of fine-scale structure. An animatable displacement model using UV displacement maps was performed in [16] to generate expression-related wrinkles. The representational realism of reconstructions was enhanced by separating reflectance and geometric detail attributes and modeling them independently in [32]. On the basis of texture, some static–dynamic decoupling mechanisms were proposed in [15], to estimate static facial details, while dynamic details were detected and predicted using a pix2pixHD network assisted by facial deformation maps. The coarse-to-fine strategy UDL [14] used UV displacement depth maps on a single channel to extend medium-scale geometry representation capability. Single-image reconstruction has evolved a lot, but realism and detail accuracy are still challenging, especially with fine-scale features. Therefore, a hierarchical model structure is introduced to fill this gap that was analyzed in this research. It allows us to refine the geometric details on different levels, as such a model attempts to reconstruct realistic 3D faces. In comparison to the UDL baseline, we extended the two-stage framework to a three-stage framework in which it is actively recommended to decouple mid- and high-frequency details in order to enhance fine-detail perception. Furthermore, two novel loss functions are added and subsequently used to achieve new global–local contour consistency and high-frequency detail recovery in the second and third parts, respectively.

### 2.2. Gabor Filter

The Gabor filter, originally introduced in [33], has been demonstrated as a reliable estimate of the receptive field of the mammalian visual cortex. Its performance on both spatial and frequency scales is strong, and it also enables joint spatial–frequency analysis, allowing direct control of frequency and orientation. Thus, due to these merits, Gabor filters have been applied in computer vision applications like fingerprint recognition [34], face recognition [35], and age or gender assessment [36]. Moreover, empirical studies have demonstrated that adding Gabor responses as network inputs has a positive effect on performance of convolutional neural networks (CNNs) [37]. Then, the Gabor jet method was devised to capture multiscale and multi-orientation Gabor responses [36]. Most of these methods apply Gabor filtering as a preprocessing step, which complicates the seamless integration by Gabor filtering into the deep architectures. Recently, attempts to embed Gabor filters directly within deep neural networks have been made [38,39,40]. However, such methods generally suffer from training instability and a high computational cost, which undermines their practical usefulness. A relatively stable approach was introduced in [41], where manipulable Gabor layers were inserted in a cascaded network to enhance scale- and orientation-based image decomposition, thereby enhancing the generalization and computational efficiency. More recently proposed here, a learning-based approach for automatic Gabor parameter optimization of fine-grained recognition tasks was introduced in [18]. By setting constraints on the trained parameters, that method adapts to find the parameter combinations most appropriate to the target task. Since the Gabor filters are shown to effectively identify detailed visual information, we believe that they can be developed to support the 3D facial geometry reconstruction. Their excellent spatial–frequency discrimination is promising for improving the prediction of high-frequency detail models as a challenge in single-image 3D face reconstruction.

## 3. Proposed Frameworks

### 3.1. Overview

**Figure 2:**
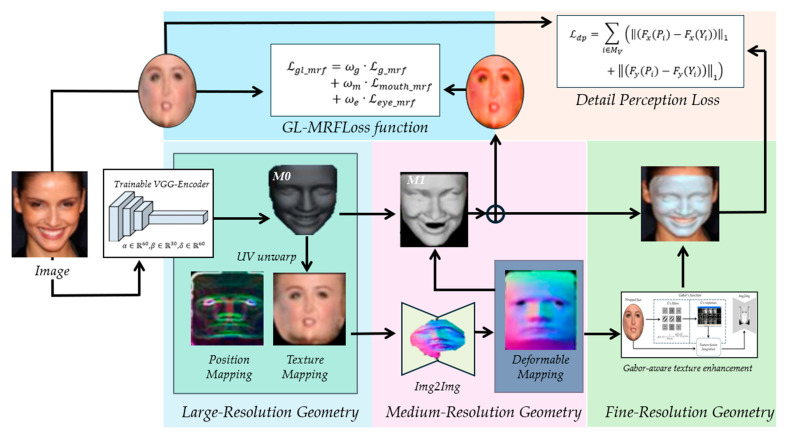
Overview of the proposed hierarchical multi-resolution self-supervised framework (HMR-Framework) for single-image 3D face reconstruction.

A hierarchical multi-resolution framework based-on self-supervised learning (HMR-Framework) is then presented to reconstruct 3D facial geometry from a single input image with multi-scale detail. The complete pipeline is depicted in Figure 2, which is divided into three successive stages. For the initial step, a trainable VGG-Encoder [42] is used to regress the parameter values of 3DMM and to recover a coarse geometric prior. In the subsequent step, a global–local Markov random field loss (GL-MRFLoss) is computed for the prediction of a three-channel vertex deformation map, which will be the model. Finally, a Gabor-aware texture enhancement module is implemented to achieve a learnable Gabor-aware texture refinement module and joint decoupling of fine-scale textures in spatial and spectral domains, in order to enhance the network better in high-range detection of delicate, high-frequency facial features. In addition, a detail perception loss (DPLoss) is used to forecast UV displacement maps enriched with finer structural features. The vertex deformation map and UV displacement map predicted from the second and third stages are subsequently combined with the coarse geometry prior to yield a fully detailed 3D facial reconstruction. Every intermediate output from the three stages is generated as 2D images, and the whole process is applied to obtain an end-to-end self-supervised learning goal where discrepancies between the 2D images and the input facial data are minimized.

### 3.2. Large-Resolution Geometry Prior Reconstruction

Since a single-image 3D facial reconstruction is an ill-posed problem, 3DMM is used as an application method for generating a rough geometric representation at the initial reconstruction stage. The initial 3D face, denoted in Figure 2, is thus regressed using a trainable VGG-Encoder [42] to obtain the 3DMM parameters.

(1) $$S=S\left(\alpha ,\beta \right)=\bar{S}+B_{id}\alpha +B_{exp}\beta$$

(2) $$T=T\left(\delta \right)=\bar{T}+B_{t}\delta ,$$

$\bar{S}$ and $\bar{T}$ are the mean shape and texture, the $B_{id}$ and $B_{t}$ are identity and texture bases, $B_{exp}$ are expression bases, and α, β, and δ the necessary parameter vectors. The latent code from just one facial image is fed through the VGG-Encoder to the parameter outputs $\alpha \in R^{60}$, $\beta \in R^{30}$, and $\delta \in R^{60}$. Like in [14], training is self-supervised, in which the reconstructed 3D face is rendered onto an input image plane, and the gap between the obtained and original image is kept as minimal as possible. The total loss is calculated as follows:

(3) $$L_{proir}={\omega_{1}\cdot L}_{pixel}+{\omega_{2}\cdot L}_{lm}+{\omega_{3}\cdot L}_{id}+{\omega_{4}\cdot R}_{param},$$

where $L_{pixel}$ is the photometric loss, $L_{lm}$ is the landmark consistency loss, $L_{id}$ is the identity-aware loss, and $R_{param}$ is a regularization term for the 3DMM parameters. Weight coefficients $\left\{\omega_{1},\;\omega_{2},\;\omega_{3},\omega_{4}\right\}$ = {1.3, 1.0, 1.5, 20.0} follow the settings found in [14].

The pipeline is organized into three progressive stages. In the first stage, a trainable encoder regresses 3D morphable model parameters to establish a coarse geometric prior. In the second stage, a three-channel vertex deformation map refines medium-scale geometric structures under global–local Markov random field supervision, ensuring structural coherence across the facial surface. In the third stage, a Gabor-aware texture enhancement module decouples spatial–frequency information to recover fine-scale details, which are further supervised by a wavelet-based detail perception loss. The outputs of all stages are fused with the geometric prior to generate a high-fidelity 3D facial reconstruction.

### 3.3. Medium-Resolution Geometry Detail Reconstruction

Although the coarse geometric prior in the first stage affords a stable global facial structure, it cannot represent medium-scale geometric features, including nasolabial folds, dimples, and facial surface variations associated with certain expressions, at the middle level of morphology. To overcome the limitation, a medium-resolution reconstruction stage is introduced through the prediction of a three-channel vertex deformation map that sharpens the coarse geometry while preserving structural consistency. It is based on a combination of photometric loss, regularization, and global–local Markov random field loss (GL-MRFLoss) and supervised. The GL-MRFLoss is deliberately designed to create structural uniformity over the face spatially by incorporating compatible global as well as local constraints. The global aspect induces a consistent shape all over the face (to avoid macro-based geometric distortions), while the local aspects emphasize high-detail parts, like the eyes and mouth, where fine structural differences show more of a visual impact. Unlike the GL-MRFLoss, it is not constructed as a monolithic constraint, and we intentionally trade-off the effects of global and regional interactions with weighted combinations, as described in Equations (6)–(8). It guarantees global facial integrity while also allowing the network to selectively enhance medium-scale details in anatomically critical locations (e.g., edges in a cross-section of the facial area). The weighting scheme is empirically established to stabilize the training sequence and to avoid over-focusing on small local areas, which would introduce geometric irregularities. This intermediate refinement phase provides the critical link between coarse geometry estimation and fine-scale texture improvement. The methodology explicitly models medium-scale deformations under strict global–local supervision and provides a well-founded geometric platform on which high-frequency details can be reconstructed.

Since the coarse geometry established in the first stage lacks mid-level structural information, a three-channel vertex deformation map is added to preserve medium-scale geometric aspects. The green highlighted module in Figure 2 depicts the image-to-image translation network [43] for reconstruction. The medium-resolution model is trained with pixel-wise photometric loss, GL-MRFLoss, and a regularization term, arriving at the following:

(4) $$L_{medium}={\omega_{p}\cdot L}_{pixel}+{\omega_{mrf}\cdot L}_{gl\_mrf}+{\omega_{r}\cdot L}_{reg},$$

with weights $\left\{\omega_{p},\;\omega_{mrf},\;\omega_{gl\_mrf}\right\}$ = {1, 1, 0.01}.

*Pixel-wise Photometric Loss*: The photometric loss calculates the L2 difference in visible pixels between the input image (I) and the rendered image ($I^{R}$):

(5) $$L_{pixel}=\frac{1}{\left|M_{V}\right|}\sum_{i\in M_{V}}{\left\|V\cdot \left(I_{i}-I_{i}^{R}\right)\right\|}_{2},$$

where ($M_{V}$) represents the visible facial region.

*Global and Local Markov Random Field Loss (GL-MRFLoss)*: Markov random field regularization has been shown to improve detail restoration for image synthesis tasks [21,44]. As a result, GL-MRFLoss aims to ensure global structural coherence and local consistency in high-detail facial regions. The global MRF loss is computed as follows from *conv*3_2 and *conv*4_2 on VGG19 following [44], increasing *conv*3_2 weights as suggested in [45]:

(6) $$L_{g\_mrf}=2\cdot L_{M}({conv3\_2}_{global})+L_{M}({conv4\_2}_{global}).$$

Local MRF losses are derived from cropped UV patches around the mouth and eyes:

(7) $$L_{m\_mrf}=2\cdot L_{M}({conv3\_2}_{mouth})+L_{M}({conv4\_2}_{mouth}).$$

and similarly for the eye region. So, the combined GL-MRFLoss is given by the following:

(8) $$L_{gl\_mrf}={\omega_{g}\cdot L}_{g\_mrf}+{\omega_{m}\cdot L}_{mouth\_mrf}+{\omega_{e}\cdot L}_{eye\_mrf},$$

with weights $\left\{\omega_{g},\;\omega_{m},\;\omega_{e}\right\}$ = {0.3, 0.5, 0.2}.

*Regularization Loss*: Regularization is also used for smooth appearance of UV normal and position maps to guarantee smoothness:

(9) $$L_{reg}=\sum_{i\in M_{V}}\omega_{n}\cdot {\left\|\left(N_{i}-N_{i}^{D}\right)\right\|}_{2}+\omega_{d}\cdot {\left\|\left(P_{i}-P_{i}^{D}\right)\right\|}_{2},$$

where $\left\{\omega_{n},\;\omega_{d}\right\}$ = {0.05, 0.01}.

### 3.4. Fine-Resolution Geometry Detail Reconstruction

#### 3.4.1. Gabor-Aware Texture Enhancement

**Figure 3:**
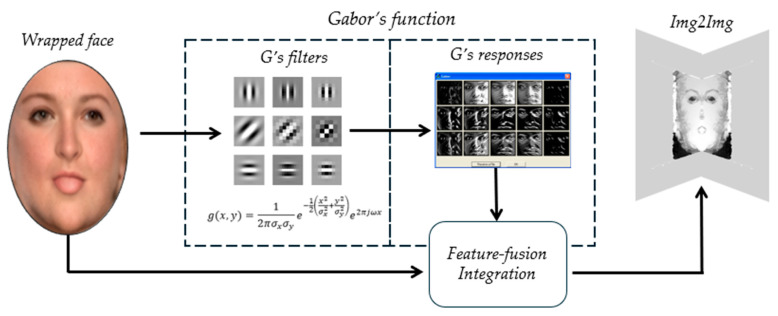
Architecture of the Gabor-aware texture enhancement module. Multiple learnable two-dimensional Gabor filters are embedded in the shallow layers of the image-to-image translation network to selectively capture high-frequency facial textures. Quadratic constraints are imposed on the Gabor parameters to ensure numerical stability and to bias learning toward small-scale, high-frequency regimes. The Gabor responses are adaptively fused with the UV texture representation using learnable weights, enabling effective integration of spatial–frequency cues for fine-detail reconstruction.

Fine-scale facial geometry from a single image is particularly challenging because medium- and high-frequency textures are strongly entangled in UV texture representations. When trained without an explicit decoupling mechanism, convolutional networks often adopt a dominance of dominant medium-scale variations, resulting in over-smoothed reconstructions and inadequate sensitivity to subtle high-frequency details, such as wrinkles and skin microstructures. To alleviate this limitation, a learnable Gabor-aware texture enhancement module has been proposed that explicitly promotes joint spatial–frequency decoupling. Gabor filters [46] are of special interest in this area because they are capable of localizing image structures simultaneously in spatial and frequency domains, thereby selectively detecting the oriented and high-frequency patterns that are characteristic of fine facial features. Multiple 2D Gabor filters are embedded in shallow layers of the image-to-image translation network in the design. Instead of pre-set parameters that are handcrafted and specific for each individual Gabor kernel, the entire optimization process is carried out through backpropagation. A quadratic constraint is imposed on the trainable parameters in order to achieve numerical stability and to move the learning toward small-scale, high-frequency regimes as in Equation (12). This restriction limits the effective parameter range in terms of optimization to avert degenerate solutions and, instead, encourages the filters to converge toward the frequency bands that are most informative for fine-detail reconstruction. It should be noted that there is no defined list of functional roles for each layer in the six-layer Gabor. Instead, layer-level specialization reveals itself implicitly through learning, so that distinct kernels learn complementary spatial–frequency responses for different scales or orientations. In practice, a multi-kernel, multi-layered design strikes a fine balance between representational capacity and computational efficiency when applied in the ablation study. Gabor filters’ output is then combined with the UV texture representation with learnable weighting coefficients to allow the adaptability of texture cues extracted from both original UV maps as well as from frequency-selective responses. This fusion strategy improves the sensitivity of the network to fine-scale geometric differences while maintaining its fidelity to the underlying facial structure. Due to the entangled nature of medium- and fine-scale facial textures, networks tend to attend to medium-level variations when performing model architectures. To alleviate this problem, we propose a learnable Gabor-aware texture enhancement module that implicitly disentangles finer-scale features, are shown in Figure 3 Through a dual restriction mechanism on learnable Gabor parameters, this module allows joint decoupling in spatial and frequency space. Embedded in the shallow layers of the image-to-image network [43], the module includes multiple learnable 2D Gabor filters together with a feature-fusion mechanism.

(10) $$g(x,y)=\frac{1}{2\pi \sigma_{x}\sigma_{y}}e^{-\frac{1}{2}\left(\frac{x^{2}}{\sigma_{x}^{2}}+\frac{y^{2}}{\sigma_{y}^{2}}\right)}e^{2\pi j\omega x},$$

with rotated coordinates:

(11) $$\begin{array}{c} x=xcos\theta \;+\;ysin\theta \\ \;y=-xsin\theta \;+\;ycos\theta . \end{array}$$

Trainable parameters $\left\{\sigma_{x}{,\sigma }_{y},\omega \right\}$ are constrained within task-specific ranges via the following:

(12) $$p=\frac{u_{p}-l_{p}e^{-P}}{1+e^{-P}},$$

providing numerical stability. Other quadratic constraints are used as restrictions on $\left\{\sigma_{x}{,\sigma }_{y},\omega \right\}$ to reduce to small-scale and high-frequency regimes.

Combining $N_{f}$ Gabor filter outputs with results from the UV texture map is a fusion mechanism as follows:

(13) $$U_{fusion}=\omega_{tex}\cdot U_{tex}+\sum_{k=1}^{N_{f}}\omega_{k}F_{g}^{k},$$

where $\left\{\omega_{tex},\;\omega_{k}\right\}$ are learnable weights optimized end-to-end.

The six-layer Gabor configuration was selected based on an empirical trade-off between representational capacity and computational stability, as demonstrated in the ablation study. Rather than assigning predefined semantic roles to individual layers, specialization emerges implicitly during training, allowing complementary orientation–frequency responses to be learned across layers. This design avoids over-parameterization while maintaining sufficient flexibility for high-frequency facial detail modeling.

#### 3.4.2. Loss Function

A composite loss function supervised the fine-resolution reconstruction phase to ensure that photometric fidelity and high-frequency details are maintained, while numerical stability is controlled. This loss equation integrates photometric loss, detail perception loss, and regularization as stated in Equation (14). A photometric loss maintains pixel-level consistency between the input image and the reconstructed image so that the recovered geometry remains visually aligned with the observed facial appearance. Yet photometric supervision is not enough to retrieve fine-scale geometric details, particularly in the presence of lighting changes and sensor noise typical of in-the-wild images. We address this limitation with a wavelet-based detail perception loss. Specifically, the Haar discrete wavelet transform is utilized to decompose both predicted and reference UV texture maps into directional high-frequency components. We employ Haar because it is computationally efficient, and sharp edge responses that are important for modeling fine facial structures, like wrinkles and skin creases, are preserved by it. It penalizes discrepancies in the high-frequency wavelet coefficients, motivating the network to adopt edge-aware texture semantics while remaining robust to low-frequency changes in illumination. Regularization is then performed to constrain the magnitude of predicted UV displacement maps, thereby minimizing noise amplification and preventing unstable surface oscillations during the process of optimization. These loss terms together offer complementary supervision, which encourages accurate fine-detail reconstruction while retaining geometric smoothness and numerical stability. Photometric loss, detail perception loss, and regularization supervise the fine-scale detail reconstruction:

(14) $$L_{fine}={\omega_{p}\cdot L}_{pixel}+{\omega_{dp}\cdot L}_{dp}+{\omega_{r}\cdot L}_{reg},$$

with weights {1.0, 1.0, 0.01}.

*Detail Perception Loss*: To retain edge-aware texture semantics, a Haar discrete wavelet transform [19] is used to compute LH and HL coefficients of both predicted UV texture and ground truth. Then, an L1 penalty is imposed:

(15) $$L_{dp}=\sum_{i\in M_{V}}\left({\left\|\left(F_{x}(P_{i})-F_{x}(Y_{i})\right)\right\|}_{1}+{\left\|\left(F_{y}(P_{i})-F_{y}(Y_{i})\right)\right\|}_{1}\right),$$

permitting the model to gain better sensitivity to high-frequency edge structures.

*Regularization Loss*: Noise sensitivity is reduced by using the following:

(16) $$L_{reg}=\sum_{i\in M_{V}}{\left\|D_{i}\right\|}_{2},$$

where $D_{i}$ indicates the UV displacement values.

## 4. Experiments

### 4.1. Implementation Details

The proposed framework is trained on the CelebA dataset, which provides a large collection of in-the-wild facial images with substantial variations in expression, illumination, and identity. CelebA is employed exclusively for training due to its scale and diversity, enabling stable self-supervised optimization without reliance on paired 3D ground truth. For evaluation, the FaceScape benchmark is adopted to assess geometric reconstruction accuracy under controlled and challenging conditions. The FaceScape-Wild subset contains synthetic renderings with varying pose angles, while the FaceScape-Lab subset provides high-resolution facial scans with accurate ground-truth geometry. This cross-dataset evaluation protocol is intentionally designed to assess the generalization capability of the proposed framework beyond the training domain.

The pose distribution in the evaluation sets spans frontal to large-angle views (up to 60°), allowing systematic analysis of reconstruction performance under increasing self-occlusion. Although occlusion and illumination variations are partially represented through pose changes and rendering conditions, it is acknowledged that the test sets do not fully cover all real-world scenarios. This limitation is explicitly discussed in Section 5.

A model was trained on the CelebA dataset [47]. A training subset containing 100,000 in-the-wild face images, of which 1000 were saved for validation, and a test one containing 19,000 images were then used. The geometry-prior reconstruction module was realized with a structure similar to VGG-Face [42]; the geometry-detail module was implemented with the image-to-image translation technique [43].

The initial learning rate in the geometry-prior reconstruction stage was equal to 0.0001 and was decayed by a factor of 0.9 for every 5000 steps. The learning rate for the geometry-detail modeling module and texture-detail modeling module was 0.00001 and decayed at a rate of 0.99 every 5000 steps. A batch size of 10 was adopted. Model optimization was performed using the Adam optimizer on a workstation with an NVIDIA GTX-5090 GPU. The geometry-detail modeling submodule was trained over 10,000 steps run period.

### 4.2. Qualitative Comparative Analysis

Comparisons were made using this method to other state-of-the-art ones, such as Deep3D [12], UDL [14], DECA [16], FaceVerse [48], EMOCA [49], and HRN [50]. Since HRN takes as ground-truth detail labels real deformation and displacement maps, it is considered a supervised method, while the other methods are weakly supervised or unsupervised. For the sake of fairness, pretrained models and official code releases were publicly available.

**Figure 4:**
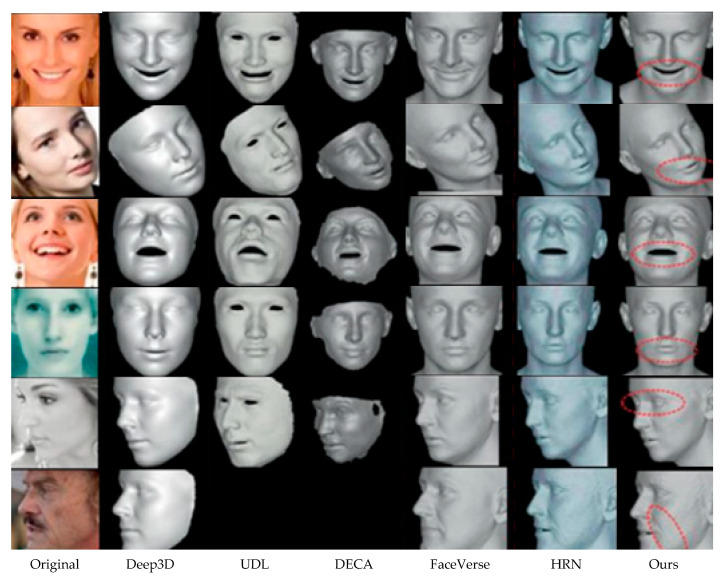
Qualitative comparison on the image test set, demonstrating fine-level detail reconstruction in the HMR-Framework, highlighted by red dashed circles.

The CelebA test set was used for all comparison experiments. Qualitative results from single-image 3D face reconstruction, as representative, are shown in Figure 4. Deep3D generates smooth geometric surfaces and no fine-scale facial detail. UDL and FaceVerse do recover a few medium-scale geometric structures, but there are large differences to the input image, especially for large-scale texture variations, and both methods show limited sensitivity for fine textures. DECA reconstructs more local features but typically produces remarkably similar features in forehead wrinkles across various identities. By contrast, the proposed approach generates high-fidelity 3D faces, which preserve medium-scale and fine-scale geometric structure together, as well as fine-scale geometric structure that corresponds quite well to those of the images for which it was trained. In particular, significant advances in the fine quality of the fine-detail reconstruction are achieved by the method compared to previous unsupervised methods. Even against supervised HRN, the proposed method reaches similar fine-scale reconstruction but has significantly better robustness under occlusion, in which HRN is very ineffective.

### 4.3. Comparison with Other Geometric Reconstruction Methods

The accuracy of geometry reconstruction was assessed by employing Chamfer Distance (CD) and Mean Normal Error (MNE), using the FaceScape benchmarking process [15], and comparing it with current approaches. The quantitative responses are presented in Table 1 on the FaceScape-Wild dataset [15], comprising 400 synthetic images with pose angle. The proposed method shows better CD performance at pose angles between 0° and 30° and is also the only method to consistently come in within the top three ranks, evaluated consistently using all metrics. The comparison on the FaceScape-Lab dataset [15] is presented in Table 2, which contains 660 high-resolution images along with ground-truth scans. This has clear leading performance in CD and MNE at 0°. Performance drops marginally between angles 30° and 60° pose, but on average, the ranking is still ranked among the best among the competing approaches.

As no quantitative metric directly evaluates fine-scale 3D facial geometry, LPIPS became an appropriate metric of this concern, as it quantifies the semantic accuracy and detail consistency, as reported in Table 3. Although LPIPS was developed specifically for real-world RGB images, its use for assessing fine-scale 3D facial detail was validated for visual similarity and semantic coherence without pixel-level alignment [51].

**Figure 5:**
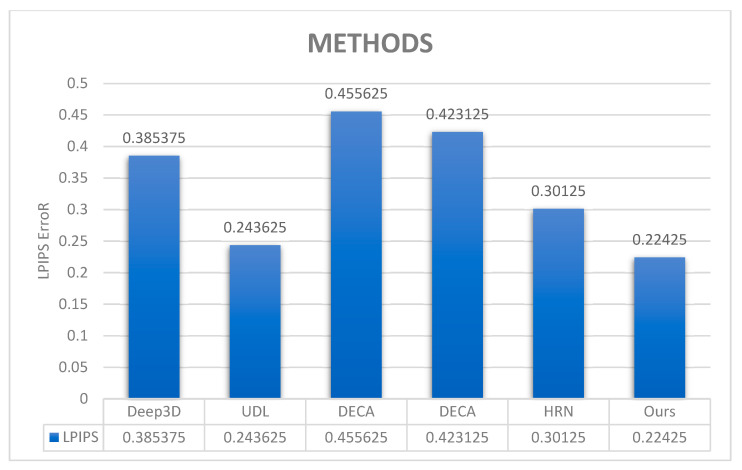
Visualization of detail consistency analysis results.

They used FaceScape-Lab images with ground-truth meshes (100 images distributed randomly). Because few of the competing models output only unrendered meshes, all the meshes of the competing methods and the ground truth were orthographically projected to 2D, aligned with the original images, and masked to retain only visible facial patches. It encoded the masked results out to latent representations, decoded back to images, and also applied LPIPS for validation. As indicated in Table 4, the proposed method has the best semantic accuracy, followed by detail consistency, which aligns with the qualitative results described in Figure 5. Although a moderate performance degradation is observed at extreme pose angles (≈60°), the proposed framework consistently ranks among the top unsupervised methods across all evaluation metrics. This behavior is primarily attributed to self-occlusion and missing texture supervision in non-visible regions, a known limitation of monocular self-supervised reconstruction. Importantly, the results demonstrate that the hierarchical design preserves competitive accuracy under moderate poses while substantially improving fine-scale detail fidelity.

### 4.4. Ablation Study

**Figure 6:**
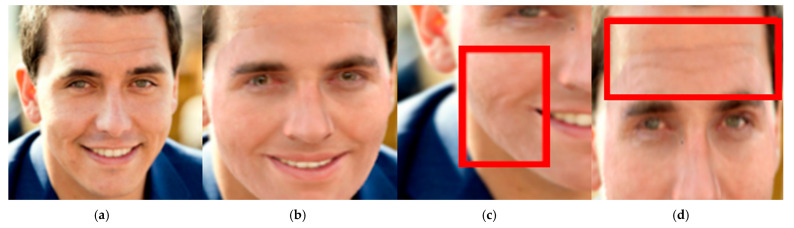
Visual comparison of the results of the framework. Column (**a**) is the original image, (**b**) is the large-scale geometry prior reconstruction result, (**c**) is the medium-scale geometry detail reconstruction result, and (**d**) is the fine-scale geometry detail reconstruction result. The red boxes emphasize regions where additional geometric details are recovered by the proposed framework at the medium-scale and fine-scale stages.

An ablation study is performed to analyze the contribution of the suggested architectural components (multi-scale hierarchical strategy, the global–local MRF loss, Gabor-aware texture enhancement module, and detail perception loss). As shown in Table 4, the reconstruction quality is significantly enhanced as each building block is added incrementally to the baseline model. LPIPS is used as a performance measurement in the ablation, as no existing metric directly quantifies perceptual consistency of fine-scale 3D facial geometry. LPIPS measures semantic similarity between the rendered reconstructions and their reference images in a learned feature space, making it an appropriate approach to evaluate high-frequency detail quality, not necessarily needing precise pixel-level correlation. Although geometric data such as Chamfer Distance (CD) and Mean Normal Error (MNE) are the two main metrics to assess overall reconstruction accuracy, LPIPS is utilized to provide perceptual fidelity of information at a fine scale. The experimental results show that the hierarchical multi-resolution approach provides the greatest performance gain, reinforcing the ability to progressively model facial geometry at different scales. The Gabor-aware module and detail perception loss improve fine-detail consistency, indicating a complementary role of these two approaches for high-frequency reconstructions. A hierarchical modeling strategy with multiple scales was applied to characterize patterns at coarse, medium, and fine dimensions, respectively, so that the facial geometry reconstruction is efficient and accurate. Figure 6 shows the representative results. The coarse-scale reconstruction stage (Figure 6b) predicts the basic 3D facial contour. The medium-scale stage (Figure 6c) improves intermediate structures, including nasolabial folds and dimples. The fine-scale stage (Figure 6d) targets fine features—such as forehead lines and crow’s feet—leading to feasible high-frequency geometry. This hierarchical method retains global structural uniformity, while also allowing for adaptable, stage-wise tuning.

A quantitative ablation study was performed by LPIPS after the validation method in [51]. The input images and the reconstructed images were coded and recoded into latent codes, decoded, and then compared with an analogue process using LPIPS. 300 CelebA test images were used in the analysis. Table 4 shows that the multi-scale hierarchical strategy reduces LPIPS error by 9% based on the baseline 3DMM. Global–local MRF loss, learnable Gabor module, and detail perception loss can be added which have an additional value of up to 0.3%, 0.7%, and 1.4%, respectively. The proposed components are confirmed to be effective with an overall 11.4% improvement from the baseline 3DMM.

Finally, to explore the effect of Gabor filter depth and kernel dimensions, further ablation experiments were completed. As summarized in Table 5, it was found that multi-kernel structures (1 × 1, 3 × 3, 5 × 5) surpass single-kernel designs using multi-kernel configurations. When six layers of Gabor filters are fitted, model performance converges; as the number of layers increases, the gains increase gradually. Thus, as the optimal computational cost vs. reconstruction accuracy trade-off, we opt for a six-layer Gabor configuration.

## 5. Discussion

In this study, the two-stage reconstruction framework provided in the baseline method [14] was generalized to a three-stage structure. For a more detailed exploration of the computational efficiency of the expanded model, a broader series of comparative experiments was performed. More precisely, 300 images were randomly sampled from the CelebA test set for inference, and the preprocessing time was omitted from this measure. Table 6 summarizes the final average computation times. Since UDL requires just two stages to obtain the final reconstruction, its computational time is reasonably low. The proposed approach only marginally differs in computation time, in comparison to HRN, which follows a three-stage system as well. These results show that a three-stage reconstruction method is somewhat less computationally efficient, although the additional computational cost remains within limits based on significant improvement in reconstruction quality. Knowledge distillation and model compression will be explored in future research to increase computational speed of the formulated model.

### 5.1. Analysis of Large-Pose Degradation

Quantitative results in Table 1 and Table 2 show a significant degradation in performance at larger pose angles (approximately 60°) where the proposed framework has higher reconstruction errors than frontal or moderate poses. This is essentially due to self-occlusion and texture omission in the non-visible parts of the facial region. When the pose becomes large, part of the face will not be visible in the input image, leading to ambiguous geometric cues and incomplete texture supervision during self-supervised training. Notably, while fully supervised approaches leverage dense ground-truth displacement maps, we propose the concept of occluded geometry to be inferred implicitly from visible cues and can introduce uncertainty near occlusion borders. This is especially visible in high-frequency areas, where texture loss and inconsistent detail propagation might exist. Such difficulties have been reported in previous monocular 3D face reconstruction work. Remedies may involve symmetry constraints, explicit UV texture completion modules, or priors based on multi-view data to increase robustness under extreme poses. Such directions are seen as promising avenues for future work.

### 5.2. Computational Efficiency and Trade-Offs

As demonstrated through the computational efficiency analysis presented in Table 6, the proposed framework takes around 6 s per inference, similar to other three-stage architectures like HRN, but slower than two-stage approaches. The increase in the run time of that process is mainly a result of the extra fine-resolution reconstruction stage and the incorporation of frequency-aware processing. It incurs these additional costs to execute, but the hierarchical architecture results in significant improvements in reconstruction accuracy, especially for fine-scale details. Both of these show a well-defined trade-off between accuracy and efficiency, with moderate increases in run time producing clear improvements in perceptual and geometric quality. Importantly, reconstruction accuracy is given priority over real-time performance in this implementation. Thus, future optimization methods such as model compression, knowledge distillation, and reduced Gabor filter depth will be expected for a greater speed of inference while not degrading the quality of the reconstruction. They will also explore the extent of GPU memory usage and parallelization efficiency to contribute to practical deplorability.

Moreover, Table 2 demonstrates a reduction in reconstruction accuracy for large-pose input. This restriction is possibly due to the lack of explicit constraints on these non-visible facial areas. Uncertainties typically occur in the gaps between the visible and occluded regions, and inadequate geometric supervision permits uneven detail generation. Future work will address this problem by including UV texture completion and prior geometric and textural cues to increase consistency regarding visual and texture detail.

## 6. Conclusions

In this work, we proposed a hierarchical multi-resolution framework supported by self-supervised learning (HMR-Framework) to model geometric facial features in different scales while also improving the recovery of fine-scale structures. The face model was divided into three hierarchical component scales, allowing structured representations of coarse, medium, and fine spatial details. A learnable Gabor-aware texture enhancement module was integrated and used to decompose fine-scale textures across spatial and frequency domains, enabling deep fine-scale processing of high-frequency facial information. Results were shown in experimental studies, which suggest that the proposed method attained competitive performance with state-of-the-art 3D face reconstruction techniques, especially for detailed fine-scale recovery. Further studies will be requiredired to improve on realistic 3D face reconstruction for occlusion but also to improve the robustness and accuracy of the proposed framework.

Experimental results on the FaceScape benchmarks (Table 1, Table 2 and Table 3) and comprehensive ablation studies (Table 4) confirm that the hierarchical multi-resolution strategy contributes most significantly to reconstruction accuracy, while the Gabor-aware texture enhancement and wavelet-based detail perception losses provide complementary gains in fine-scale detail recovery.

## Figures and Tables

**Table 1:** Quantitative evaluation of FaceScape-Wild dataset.

Methods	0–5°		5–30°		30–60°	
	CD	MNE	CD	MNE	CD	MNE
DFDN	3.702	0.091	3.307	0.092	7.313	0.130
DF2Net	2.953	0.122	2.441	0.129	6.625	0.159
UDL	2.353	0.092	3.287	0.094	4.294	0.109
FaceScape	2.842	0.087	3.178	0.094	4.045	0.109
SADRNet	3.268	0.114	3.617	0.074	6.488	0.120
LAP	4.238	0.093	4.524	0.082	6.010	0.100
DECA	2.913	0.081	2.664	0.080	2.912	0.093
EMOCA	2.709	0.090	2.714	0.099	2.943	0.101
HRN	2.529	0.086	2.612	0.115	2.15	0.080
HMR-Framework	2.225	0.087	2.488	0.083	3.343	0.103

**Table 2:** Quantitative analysis of FaceScape-Wild dataset.

Methods	0°		30°		60°	
	CD	MNE	CD	MNE	CD	MNE
DFDN	5.350	0.138	8.390	0.165	29.540	0.350
DF2Net	5.600	0.190	9.550	0.250	N/A	N/A
UDL	2.760	0.115	6.680	0.154	7.040	0.209
FaceScape	4.010	0.113	6.090	0.149	5.850	0.182
SADRNet	5.320	0.136	8.840	0.171	8.860	0.185
LAP	5.340	0.140	9.260	0.186	10.880	0.244
DECA	4.130	0.116	5.180	0.125	5.250	0.134
EMOCA	3.090	0.108	4.060	0.117	5.380	0.124
HRN	2.950	0.106	4.700	0.118	5.290	0.118
HMR-Framework	2.660	0.112	6.290	0.133	6.040	0.188

**Table 3:** Detailed consistency analysis.

Methods	Deep3D	UDL	DECA	DECA	HRN	HMR-Framework
**LPIPS**	0.385375	0.243625	0.455625	0.423125	0.30125	0.22425

**Table 4:** Ablation study reporting LPIPS results for different component configurations in the HMR-Framework.

Base Model	MulHi	$L_{gl\_mrf}$	Gabor	$L_{dp}$	**LPIPS**
√	☐	☐	☐	☐	0.1037
√	√	☐	☐	☐	0.0944
√	√	√	☐	☐	0.0941
√	√	√	√	☐	0.0934
√	√	√	√	√	0.0921

**Table 5:** Ablation study comparing the number of Gabor filters and model accuracy.

Convolution Kernel Size		Number of Gabor Filters	LPIPS
	1×1, 1×1, 1×1	×1	0.0941
	3×3, 3×3, 3×3	×1	0.0943
	5×5, 5×5, 5×5	×1	0.0938
	1×1, 3×3, 5×5	×1	0.0934
⇨	1×1, 3×3, 5×5	×2	0.0921
	1×1, 3×3, 5×5	×3	0.0925
	1×1, 3×3, 5×5	×4	0.0919
	1×1, 3×3, 5×5	×5	0.0922

⇨ We selected the most optimized solution.

**Table 6:** Computational efficiency analysis.

Methods	UDL	HRN	HMR-Framework
**Times (s)**	5.3808	5.8664	6.0192

## Data Availability

The data presented in this study are openly available in CelebA dataset at https://mmlab.ie.cuhk.edu.hk/projects/CelebA.html, (accessed on 16 March 2023). The FaceScape dataset used for evaluation is available at https://facescape.nju.edu.cn/, (accessed on 16 March 2023) subject to the dataset’s license agreement.
